# SNAI1-dependent upregulation of CD73 increases extracellular adenosine release to mediate immune suppression in TNBC

**DOI:** 10.3389/fimmu.2022.982821

**Published:** 2022-09-09

**Authors:** Meriem Hasmim, Malina Xiao, Kris Van Moer, Akinchan Kumar, Alexandra Oniga, Michel Mittelbronn, Caroline Duhem, Anwar Chammout, Guy Berchem, Jean Paul Thiery, Marianna Volpert, Brett Hollier, Muhammad Zaeem Noman, Bassam Janji

**Affiliations:** ^1^ Tumor Immunotherapy and Microenvironment Group, Department of Cancer Research, Luxembourg Institute of Health (LIH), Luxembourg, Luxembourg; ^2^ National Center of Pathology (NCP), Laboratoire Nationale de Santé (LNS), Luxembourg, Luxembourg; ^3^ Department of Hemato-Oncology, Centre Hospitalier du Luxembourg, Luxembourg, Luxembourg; ^4^ Department of Oncology, Faculty of Medicine, University of Aleppo, Aleppo, Syria; ^5^ Department of Oncology, Aleppo Hospital University, Aleppo, Syria; ^6^ Guangzhou Laboratory, Guangzhou, China; ^7^ Australian Prostate Cancer Research Centre-Queensland (APCRC-Q), School of Biomedical Sciences, Faculty of Health, Princess Alexandra Hospital, Translational Research Institute, Brisbane, QLD, Australia

**Keywords:** CD73, SNAI1, epithelial-to-mesenchymal transition, adenosine, anti-tumor immune response, immunotherapy, breast cancer, immune checkpoints

## Abstract

Triple-negative subtype of breast cancer (TNBC) is hallmarked by frequent disease relapse and shows highest mortality rate. Although PD-1/PD-L1 immune checkpoint blockades have recently shown promising clinical benefits, the overall response rate remains largely insufficient. Hence, alternative therapeutic approaches are warranted. Given the immunosuppressive properties of CD73-mediated adenosine release, CD73 blocking approaches are emerging as attractive strategies in cancer immunotherapy. Understanding the precise mechanism regulating the expression of CD73 is required to develop effective anti-CD73-based therapy. Our previous observations demonstrate that the transcription factors driving epithelial-to-mesenchymal transition (EMT-TF) can regulate the expression of several inhibitory immune checkpoints. Here we analyzed the role of the EMT-TF SNAI1 in the regulation of CD73 in TNBC cells. We found that doxycycline-driven SNAI1 expression in the epithelial -like TNBC cell line MDA-MB-468 results in CD73 upregulation by direct binding to the CD73 proximal promoter. SNAI1-dependent upregulation of CD73 leads to increased production and release of extracellular adenosine by TNBC cells and contributes to the enhancement of TNBC immunosuppressive properties. Our data are validated in TNBC samples by showing a positive correlation between the mRNA expression of CD73 and SNAI1. Overall, our results reveal a new CD73 regulation mechanism in TNBC that participates in TNBC-mediated immunosuppression and paves the way for developing new treatment opportunities for CD73-positive TNBC.

## Introduction

Breast cancer is the most frequently diagnosed malignancy in women. Triple-negative is a subtype of breast cancer (TNBCs) that does not express estrogen receptors, progesterone receptors, and human epidermal growth factor receptor-2/neu (HER-2). TNBC accounts for 15% of all breast cancers exhibiting high probability of disease relapse and the highest mortality rate among breast cancer subtypes (1). TNBC patients do not benefit from hormonal therapy or HER-2 blockade, making conventional chemotherapy the only established therapeutic option which does not prevent high recurrence rates, acquired resistance, and metastasis [2].

Compared to other breast cancer subtypes, TNBCs have enhanced intra-tumoral T cell infiltration and a higher mutational burden (1, 2). Therefore, TNBCs have an increased potential to generate immunogenic mutations and are considered eligible tumors for immune checkpoint inhibition-based therapy. Recent clinical trials in TNBC patients based on PD-1/PD-L1 blockade revealed an overall response rate of 20% (3). Despite this promising clinical response, most enrolled patients showed little or no therapeutic benefit, fostering the need for alternative immunotherapeutic approaches.

The ectonucleotidase CD73 is an attractive target in cancer immunotherapy (4). CD73 is involved in generating extracellular adenosine (ADO), a potent immunosuppressive molecule for both innate and adaptive immunity (4, 5). Indeed, ADO inhibits the anti-tumor function of T and Natural Killer (NK) cells and enhances the immunosuppressive function of T regulatory cells and tumor-associated macrophages (TAM).

CD73 is upregulated in many cancer types, including breast cancer. CD73 expression is negatively regulated by estrogen receptor signaling (6). Therefore, the absence of estrogen receptors in TNBCs could contribute to CD73 expression. In addition, analysis of CD73 expression in TNBC patients shows that high CD73 is associated with decreased overall and disease-free survival and increased resistance to conventional chemotherapy (7, 8).

The molecular mechanisms involved in regulating CD73 expression are not yet fully understood. It is reported that the transcription factor Hypoxia-inducible factor (HIF)-1 is involved in directly activating CD73/NT5E expression (9, 10). However, no data are available on whether and how CD73 is regulated during tumor progression and metastatic spread.

Epithelial-to-mesenchymal transition (EMT) is a process whereby epithelial cells acquire motile and invasive mesenchymal features. EMT is driven by a series of EMT-inducing transcription factors (EMT-TFs). EMT in tumor cells is associated with increased aggressiveness, drug resistance, and immune escape. We have previously demonstrated that both PD-L1 and CD47 inhibitory immune checkpoints are upregulated in human mesenchyma -like breast cancer cell lines by mechanisms involving the EMT-TFs ZEB1 or SNAI1 (11, 12). In line with our previous work and considering the key functions of the EMT-TF SNAI1 in TNBC aggressiveness (13), we investigated the role of SNAI1 in the modulation of CD73 expression in TNBC cells and the functional impact of such modulation on the immunosuppressive properties of TNBC cells.

## Materials and methods

### Cell culture, treatment and transfection

Human TNBC cell lines MDA-MB-231 and MDA-MB-468 were purchased from DSMZ (Braunschweig; Germany). MDA-MB-468-iSNAI1 and MDA-MB-468-iGFP cells stably expressing doxycycline-inducible SNAI1 and GFP, respectively, were provided by Dr. Brett G. Hollier (Brisbane, Queensland, Australia).

MDA-MB-231 cells were cultured in RPMI 1640-GlutaMAX™, 10% FBS, and 1% Penicillin-Streptomycin. MDA-MB-468 cells were cultured in DMEM-HighGlucose-Glutamax, 10% FBS, and 1% Penicillin-Streptomycin. The NK92-MI cell line was cultured in RPMI 1640-GlutaMAX™, 10% FBS, 10% Horse Serum (ATCC), and 1% Penicillin-Streptomycin. The mouse TNBC cell line Py8119 was purchased from ATCC and was cultured in F-12K Medium, 5% FBS.

NK cells from healthy donors (NKD) were obtained from fresh apheresis products after Ficoll-Paque Plus centrifugation (GE Healthcare) and purification using a human NK Cell Isolation Kit (Miltenyi Biotec). Purified NKD were cultured in RPMI 1640-GlutaMAX™, 10% pooled human serum (Jacques Boy), 5% FBS, 1% Penicillin-Streptomycin, and IL-2 (150 UI/ml (Immunotools). All cells were grown at 37°C under humidified conditions and 5% CO_2_ and routinely tested for Mycoplasma free (MycoAlert Detection Kit; Lonza).

For SNAI1 induction, MDA-MB-468-iSNAI1 and MDA-MB-468-iGFP cells were seeded 24h before Doxycycline (Dox) (D9891, Sigma-Aldrich-Merck) treatment. The indicated doses of Dox were added every 48 h in fresh medium for 5 days, and cells were harvested on day 6. For rhEGF (#E9644; Sigma) treatment, MDA-MB-468 cells were seeded 24 h before rhEGF treatment. rhEGF (50 ng/ml) were added every 48 h in a fresh medium containing 0.5%FBS for 5 days, and cells were harvested on day 6.

Control CRISPR/Cas9 and SNAI1 CRISPR/Cas9 plasmids were obtained from Santa Cruz Biotechnology and transfected into Py8119 cells according to manufacturer’s protocol.

### Antibodies

The following antibodies for Western blot, confocal, and ChIP were from Cell Signaling: anti-SNAIL (#3879S), anti-ZEB1 (#D80D3), anti-E-cadherin (#24E10), anti-Vimentin (#D21H3) XP^®^. For others: Anti-β-Actin−Peroxidase (A3854; Sigma-Aldrich-Merck), Alexa 488-conjugated secondary antibody (1/400, Molecular Probes), Actin-Stain 488 Phalloidin (1/400; Cytoskeleton, Inc.). FACS and ImageStream antibodies were as follows: CD73-PE antibody (344004; 1:100, Biolegend), Alexa 633-conjugated secondary antibody (1/500; Molecular Probes), Ki67-PE (151210; 1:100; Biolegend).

### Quantitative real-time PCR

Total RNA was extracted from cell lines using the Nucleospin RNA Plus Kit (Macherey-Nagel). Total RNA from 12 TNBC patients was purchased from Origene (CR561562, CR561706, CR561397, CR562540, CR562125, CR560441, CR560325, CR561546, CR561196, CR561161, CR561083, CR560707). RNA was reverse-transcribed using the Maxima First-Strand cDNA Synthesis Kit (Thermo Fischer Scientific) and amplified by qPCR using the Power SYBR Green PCR Master Mix (Eurogentec). mRNA levels of genes of interest were normalized to housekeeping 18S mRNA levels.

### Western blotting

Adherent cells were lysed on ice in 62.5 mM Tris-HCl [pH 6.8], 2% w/v SDS, 10% glycerol, and 1× protease inhibitor cocktail (Thermo Fischer Scientific). Protein extracts were separated by SDS-PAGE and transferred onto nitrocellulose membranes (VWR). Primary antibodies were incubated overnight at 4°C and visualized using peroxidase-conjugated secondary antibodies (DAKO) and Western Lightning Ultra (Perkin Elmer). Blots were scanned and processed using ImageJ software.

### Flow and imaging cytometry

Cells were harvested in 10 mM EDTA (Invitrogen). Surface staining was done at 4°C for 30 min. Intracellular staining was done with Cyto-Fast™ Fix/Perm Buffer Set (426803; Biolegend). Ki67 staining in NK cells was performed following ice-cold ethanol (70%) fixation. Dead cells were excluded using Live/Dead staining Kits (L34976; Thermo Fischer Scientific) or BD Via-Probe™ Cell Viability Solution (555815; Becton Dickinson). Samples were processed on a CytoFLEX flow cytometer and analyzed using CytExpert software. For imaging cytometry, cells were fixed on ice for 20min in 1% PFA after staining, resuspended in 2%FBS at a concentration of 2×10^7^ cells/ml, and processed on ImageStreamX MKII (EMD Millipore) and analyzed using IDEA software.

### ChIP assay

ChIP was performed on MDA-MB-468-iSNAI1 lysates using the SimpleChIP Enzymatic Chromatin IP kit (#9005; Cell Signaling). EMT-TF binding to E-box in the proximal region of human E-cadherin promoter was used as a positive control (14). SYBR Green RT-qPCR was performed using primers described in **Table S1**.

### Extracellular adenosine measurement

Cell-conditioned media were collected and centrifuged at 2000 rpm, 4°C, for 15 min to remove cellular debris. Extracellular ADO concentration was determined based on a standard curve using Adenosine Assay Kit (K237-100; Biovision). Adenosine levels were determined according to the number of cells counted at the end of the experiment, as previously reported (9). The CD73 inhibitor APCP (M3763; Sigma) was added at 100 µM simultaneously with Dox.

### Adenosine analog (CADO) treatment and immune-cytotoxicity assays

CADO (C5134; Sigma-Aldrich-Merck) was used at 5 µM or 10 µM. NK cells were treated with CADO every 2 days and harvested on days 4 or 6. Cytotoxicity assays were performed as previously described (15).

### Confocal analysis

Cells were fixed for 20 min in 4% PFA at room temperature (RT), permeabilized with 0.1% Triton (10 min, RT), and blocked for 1 h with 10% FBS at RT. Antibodies were incubated for 1 h at RT, and nuclei were stained with DAPI for 5 min at RT. Images were acquired on confocal LSM880 Airy (Carl Zeiss). Scale bars were determined using ZEN 3.0 (blue edition) software.

### *In silico* TNBC data mining

TNBC patients (n = 258) from the METABRIC dataset were downloaded from cbioportal v3.4.12. TNBC patients were defined based on their negative expression of ER, PR, and HER2. mRNA levels of *NT5E* (CD73), *SNAI1* (SNAI1), *VIM* (Vimentin), and *CDH1* (E-Cadherin) were extracted, and the co-expression was defined on cBioPortal (https://www.cbioportal.org/). The associations between *NT5E*/CD73, *SNAI1*, *VIM*, and *CDH1* expressions were analyzed by using the Pearson correlation test.

### Statistical analyses

All statistical analyses were done in GraphPadPrism v8.0. Unpaired Student’s t-tests or Mann-Whitney tests were used depending on whether the data presented Gaussian distribution with *P* < 0.05 considered significant. The Pearson correlation coefficient (two-tailed confidence interval of 95%) was used to assess the correlation between EMT-TF and CD73.

## Results and discussion

### Upregulation of CD73 in TNBC cell lines is associated with mesenchymal features

**Figure 1:**
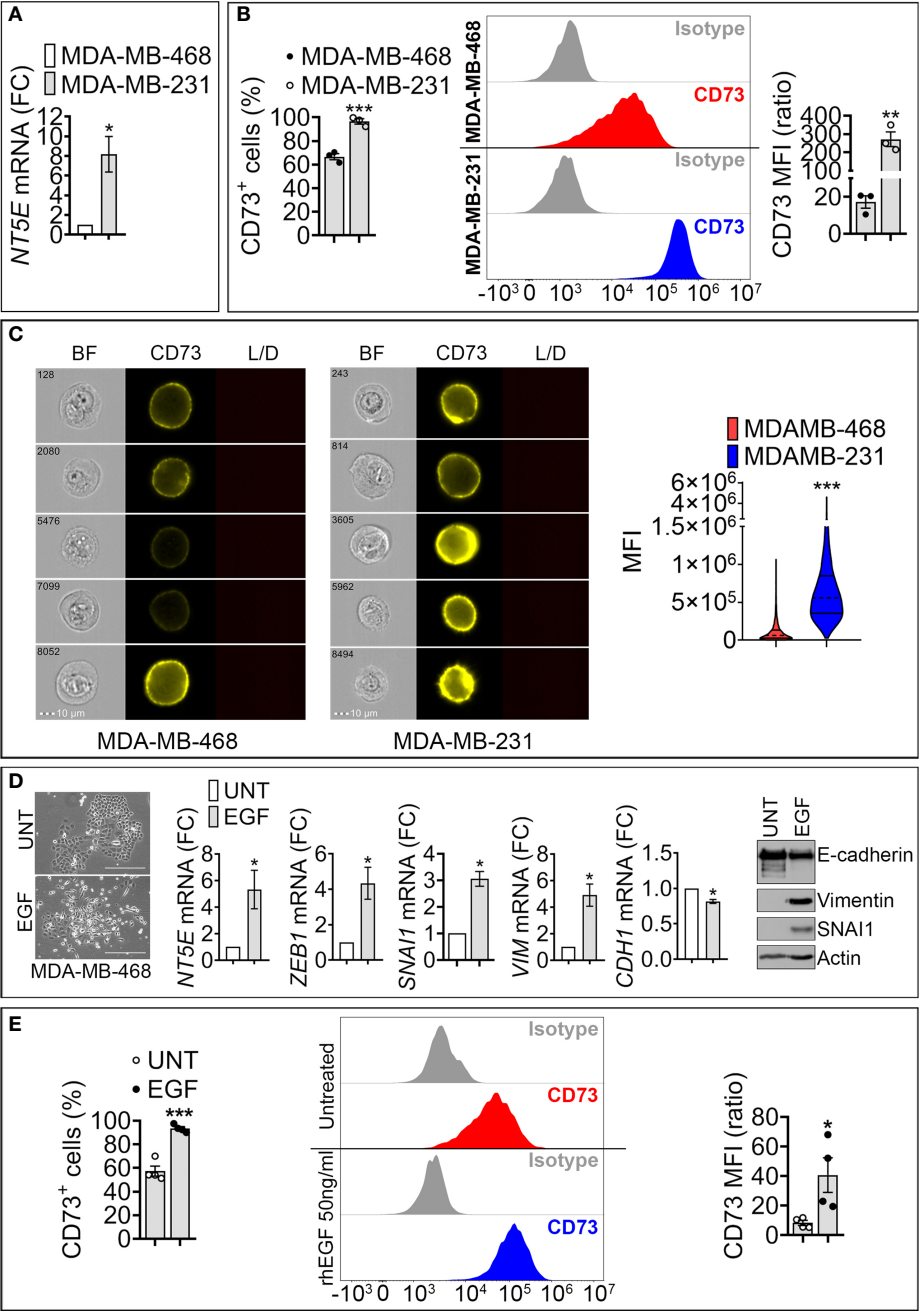
Expression of *NT5E*/CD73 mRNA and protein in mesenchymal-like MDA-MB-231 and epithelial like MDA-MB-468 TNBC cells. **(A)** RT-qPCR measurement of *NT5E* mRNA in mesenchymal-like MDA-MB-231 and epithelial-like MDA-MB-468 TNBC cell lines. NT5E expression was calculated relative to MDA-MB-468 cells. Bars represent means from four independent experiments ± SEM; *P < 0.05 calculated by Mann Whitney. **(B)** Flow cytometry analysis of cell surface CD73 in TNBC cells. *Left panel:* percentage of CD73-positive (CD73^+^) cells; *Middle panel*: representative FACS histograms of indicated cells stained with control isotype or anti-CD73 antibody. *Right panel:* mean fluorescence intensity (MFI) of cell surface CD73 in MDA-MB-468 and MDA-MB-231 cells. Bars represent means from three independent experiments ± SEM, **P <0.01, ***P < 0.001 calculated by unpaired t-test. **(C)** CD73 cell surface expression in MDA-MB-468 and MDA-MB-231 cells acquired by imaging cytometry*. Left panels:* representative images for each cell line acquired on brightfield (BF), CD73-PE, and live/dead (L/D) channels. The scale bar and event number are shown. *Right panel:* Violin plot quantification of CD73 mean fluorescence intensity (MFI) acquired by imaging cytometry in MDA-MB-468 and MDA-MB-231 cells. Results are the average of 10^4^ acquisitions, ***P < 0.001 by unpaired t-test. **(D)** *Left panels:* Morphology of untreated (UNT)- and EGF (EGF)-treated MDA-MB-468 cells. Bar: 300μm. *Middle panels:* mRNA expression of CD73 and EMT markers (SNAI1, ZEB1, VIM and CDH1) in MDA-MB-468 cells treated with rhEGF (50ng/ml for 6 days). The expression level of each gene in treated (EGF) cells was calculated relative to untreated (UNT) cells. Bars represent means from four independent experiments ± SEM; *P < 0.05 calculated by Mann Whitney. *Right panel*: Representative Western-blot showing the protein expression of E-cadherin, SNAI1, Vimentin in (UNT)- and EGF (EGF)-treated MDA-MB-468 cells. Actin was used as a loading control. **(E)**. Flow cytometry quantification of surface CD73. *Left panel:* Percentage of CD73 positive MDA-MB-468 cells treated as described in **(D)**. *Middle panel*: representative FACS histograms of untreated or rhEGF-treated MDA-MB-468 cells stained with isotype or anti –CD73 antibody. *Right panel:* Mean fluorescence intensity (MFI) of cell surface CD73 in MDA-MB-468 treated as described in **(D)**. Bars represent means from four independent experiments ± SEM; *P < 0.05, and ***P < 0.001 are calculated by unpaired t-test.

We analyzed the expression of CD73 in epithelial -like MDA-MB-468 and mesenchymal -like MDA-MB-231 TNBC cell lines. MDA-MB-468 cells express high levels of the epithelial marker E-cadherin, whereas the mesenchymal -like MDA-MB-231 cells express high levels of the mesenchymal markers ZEB1 and Vimentin (**Figure S1**). Using RT-qPCR, flow cytometry, and imaging cytometry, we measured *NT5E* (encoding CD73) mRNA and CD73 surface expression in both cell lines. We found that *NT5E* mRNA and CD73 cell surface protein are upregulated in mesenchymal MDA-MB-231 cells as compared to epithelial MDA-MB-468 (**Figures 1A–C**).

To assess the impact of EMT on CD73 expression, we used recombinant human epidermal growth factor (EGF), a potent inducer of EMT in epithelial MDA-MB-468 cells (16). Treatment of MDA-MB-468 cells with EGF for 6 days induced morphological changes consisting of a loss of cell-cell contacts and the acquisition of an elongated mesenchymal phenotype (**Figure 1D**, left panels). EGF also increased the expression of the mesenchymal markers SNAI1, ZEB1, and Vimentin and decreased the epithelial marker E-cadherin (**Figure 1D**, middle and right panels). This transition was associated with a significant increase in *NT5E* mRNA (**Figure 1D**, middle panel) and in the percentage (%) of CD73 positive MDA-MB-468 cells, as well as CD73 mean fluorescence intensity (MFI) (**Figure 1E**). Together, these results suggest that acquiring mesenchymal characteristics in TNBC cells is associated with increased CD73 expression at transcriptional and protein levels.

### The EMT-TF SNAI1 is involved in the upregulation of CD73 in MDA-MB-468

*SNAI1* is relatively more expressed than other EMT-TFs in TNBC (13). To assess the potential regulation of CD73 by SNAI1 in TNBC cells, we used MDA-MB-468 cells expressing Dox-inducible SNAI1 (MDA-MB-468-iSNAI1 cells). We first determined the appropriate Dox concentration to induce SNAI1 in MDA-MB-468-iSNAI1 cells. Using increasing Dox concentrations (0.25, 0.5, and 1 µg/ml), we showed a consistent and dose-dependent induction of *SNAI1* and *NT5E* mRNA (**Figure S2A**), indicating the potential role of SNAI1 in the transcriptional activation of *NT5E* expression.

As the maximum increase in *NT5E* expression was observed at 1 µg/ml Dox, we considered this concentration for subsequent experiments. It should be highlighted that MDA-MB-468 expressing Dox-inducible GFP (MDA-MB-468-iGFP), used as control, did not show *SNAI1* or *NT5E* induction following Dox treatment, thus ruling out any off-target effect of Dox on SNAI1 and/or NT5E expression (data not shown).

**Figure 2:**
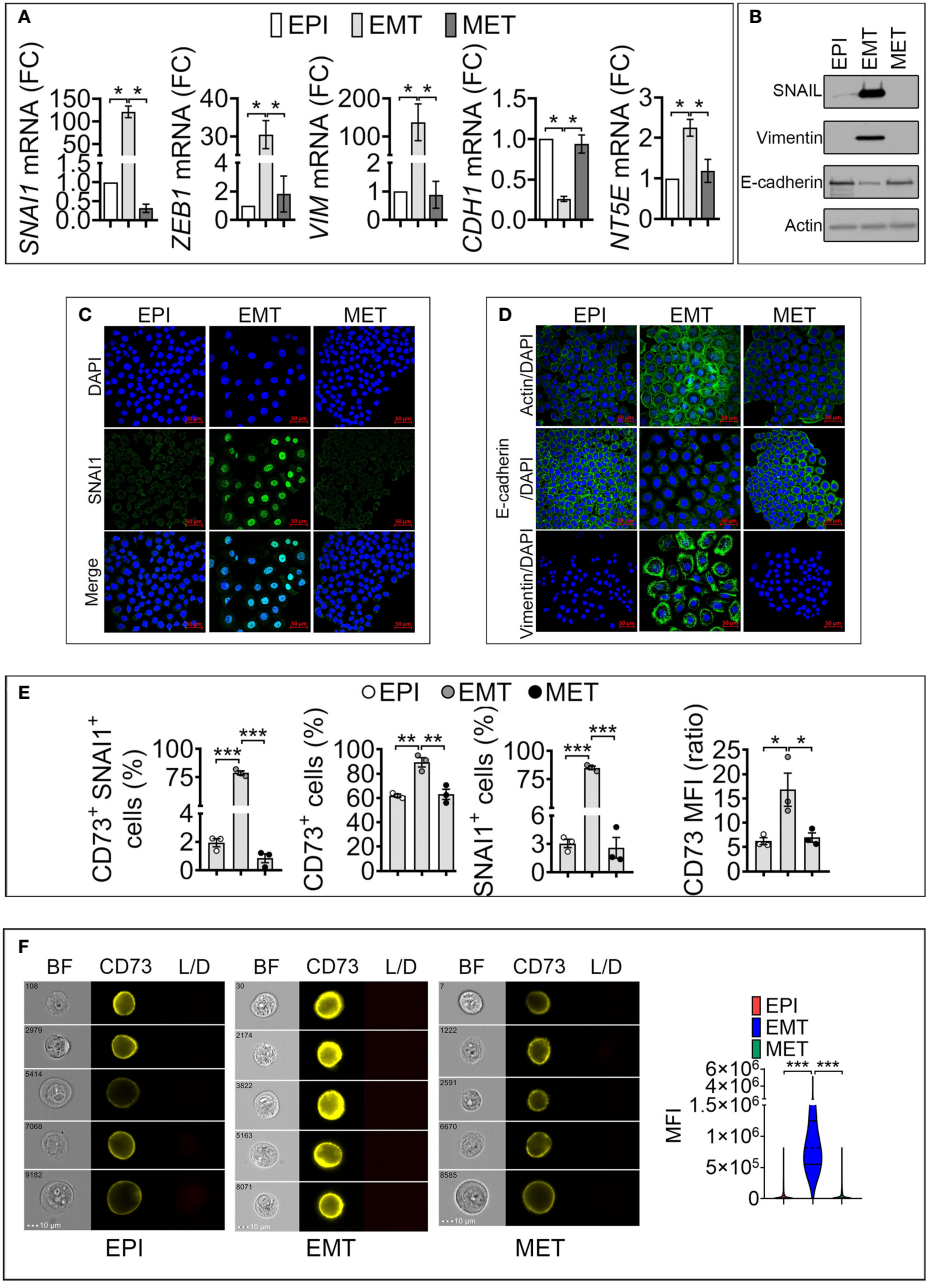
Induction of SNAI1 in TNBC MDA-MB-468-iSNAI1 cells upregulates NT5E/CD73 mRNA and protein expression. **(A)** mRNA expression of *SNAI1*, *ZEB1, VIM*, *CDH1 NT5E* and *NT5E* in EPI, EMT, and MET conditions. The expression of each gene in EMT and MET conditions was calculated relative to the EPI condition. Bars represent means from four independent experiments ± SEM (*P < 0.05 calculated by Mann Whitney). **(B)** Representative Western-blot showing the protein expression of SNAI1, Vimentin, and E-cadherin in MDA-MB-468 cells cultured under EPI, EMT, and MET conditions. Actin was used as a loading control. **(C, D)** Immunofluorescence images of SNAI1 **(C)**, Actin, E-cadherin, and Vimentin **(D)** staining (in green) in MDA-MB-468 cells cultured under EPI, EMT, or MET conditions. Nuclei are stained with DAPI (blue). The images shown are representative of two independent experiments. Scale bar: 50 µm. **(E)** Flow cytometry analysis of the percentage (%) of MDA-MB-468 cells cultured under EPI, EMT, and MET conditions that are positive for both CD73/SNAI1 (CD73+ SNAI1+) and either CD73 (CD73+) or SNAI1 (SNAI1+). CD73 MFI is reported. Bars represent means from three independent experiments ± SEM (*P < 0.05, **P <0.01, ***P < 0.001 by unpaired t-test). **(F)** CD73 surface expression in MDA-MB-468 cultured under EPI, EMT, and MET conditions by flow imaging cytometry. *Left panels:* representative images of 10^4^ acquisitions for each condition on brightfield (BF), CD73 and live/dead (L/D) channels. The scale bar and event number are shown. *Right panel:* Violin plot quantification of CD73 mean fluorescence intensity (MFI) in MDA-MB-468. Results are the average of 10^4^ events for each condition. (***P < 0.001 calculated by unpaired t-test).

To elucidate the link between SNAI1 and *NT5E*/CD73 expression, we treated cells with Dox for 5 days to induce EMT in epithelial cells (designated as EPI). We next removed Dox from the culture medium of resulting mesenchymal-like cells (designated as EMT) to revert EMT and re-acquire an epithelial-like phenotype, designated as mesenchymal to epithelial transition (MET). Our data (**Figures 2A** **, B**) show that driving EMT in MDA-MB-468-iSNAI1 cells was associated with an increase in *SNAI1*, *ZEB1*, *VIM*, and *NT5E* and a decrease in *CDH1* expression. Immunofluorescence staining showed the acquisition of mesenchymal features under these experimental conditions, as evidenced by the nuclear accumulation of SNAI1 protein, actin microfilament remodeling, Vimentin upregulation, and E-cadherin downregulation (**Figures 2C** **, D**). All these events, observed by inducing EMT, were abrogated on day 17 following Dox removal leading to a MET switch (**Figures 2A** **–D**).

To evaluate whether the regulation of CD73 and SNAI1 following EMT and MET occurred in the same cell populations, by flow cytometry, we quantified MDA-MB-468-iSNAI1 positive cells for both CD73 and SNAI1 under EPI, EMT and MET conditions. We showed that under EPI conditions, only 2% of cells were positive for both CD73 and SNAI1 (CD73^+^ SNAI1^+^). The percent of CD73^+^ SNAI1^+^ cells significantly increased to 80% under EMT and subsequently decreased to almost 1% under MET conditions (**Figure 2E** and **Figure S2B**). Our results reported in **Figure 2E**, showing the regulation of CD73 under EPI, EMT, and MET conditions, were reproduced by imaging cytometry (**Figure 2F**). Together, our results demonstrate a positive regulation of *NT5E*/CD73 following SNAI1 induction at the transcriptional and protein level in MDA-MB-468-iSNAI1 cells. The regulation of *NT5E*/CD73 by SNAI1 is further confirmed using the additional mouse TNBC cell line Py8119 displaying several mesenchymal features (absence of E-cadherin expression, expression of the EMT-transcription factors SNAIL1 and ZEB1, and higher expression of N-Cadherin and Vimentin) compared to the epithelial-like Py230 cell line. Both cell lines are derived from MMTV-PyMT transgene-induced mammary tumors (17) (**Figure S2C**). Using CRISPR/Cas9 technology, we generated Py8119 cells expressing a truncated non-functional SNAIL1 (Py8119 Del SNAIL) (**Figure S2D**). Compared to control cells, we showed that Py8119 Del SNAIL cells expressed significantly lower levels of *NT5E* mRNA (**Figure S2E**) and protein (**Figure S2F**).

### CD73/*NT5E* is a direct target of SNAI1 in MDA-MB-468 cells, and its expression positively correlates with SNAI1 in TNBC patients

**Figure 3:**
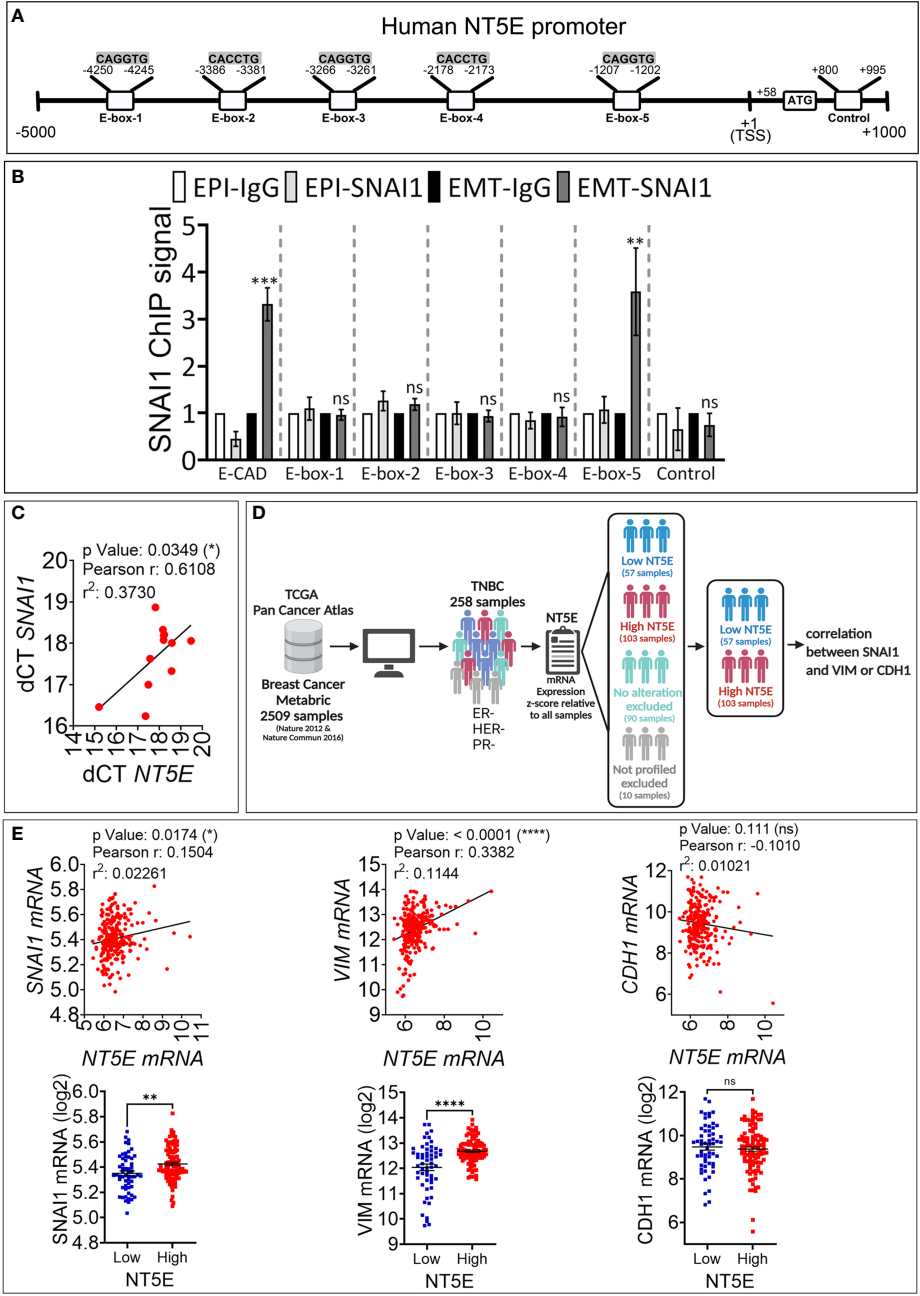
SNAI1 directly regulates CD73 expression in human TNBC MDA-MB-468-iSNAI1 cells. **(A)** Schematic representation of the different E-boxes identified *in silico* in the human CD73 promoter (CD73 mRNA, NCBI Reference Sequence: NM_002526). The transcription Start Site (TSS) and the ATG start codon are reported at positions +1 and +58, respectively. The control motif (+800 to +995) corresponds to a region containing no E-box and is used as a negative control. **(B)** ChIP was performed on MDA-MB-468-iSNAI1 cells cultured under EPI or EMT conditions using anti-SNAI1 antibodies followed by five pairs of primers flanking the identified E-boxes (E-box-1-5) or primers flanking control region. E-cadherin (E-CAD) primers were used as a positive control. For each gene, the RT-qPCR signals were normalized to control IgG. SNAI1 ChIP signal was reported as fold enrichment over IgG control. Two individual experiments (done in triplicate) were performed (*P < 0.05, **P <0.01, ***P < 0.001 calculated by unpaired t-test). **(C)** Correlation between *NT5E*/CD73 and *SNAI1 i*n tumor mRNA from 12 TNBC patients. Pearson correlation coefficient (r) and P-value are shown. **(D)** Analysis process of METABRIC dataset. **(E)** Correlation between *NT5E*, *SNAI1*, *VIM*, and *CDH1* gene expression in TNBC patients from METABRIC dataset (n = 258). For each association: Upper panel: correlation between *NT5E*/CD73 and the indicated gene. Pearson coefficients and P values are shown. Lower panel: TNBC samples were separated according to *NT5E*/CD73 expression level to form high (Z score ≥ +0.5) and low (Z score ≤-0.5) *NT5E*/CD73 groups. In each group. *SNAI1*, *VIM*, and *CDH1* gene expression were evaluated. Dots represent mRNA level ± SEM (**P <0.01, ****P < 0.0001 calculated by unpaired t-test). ns, not significant.

To assess whether CD73 is a direct target of SNAI1, we analyzed *in silico* the presence of the putative SNAI1 binding motifs CAGGTG and CACCTG, called E-boxes, in the proximal promoter of the *NT5E* gene. By using the Eukaryotic Promoter Database (Swiss Institute of Bioinformatics) and fuzznuc (EMBOSS explorer) software, we identified three CAGGTG (E-box 1, 3, 5) and two CACCTG (E-box 2, 4) E-boxes in the human *NT5E* proximal promoter (**Figure 3A**).

We next performed ChIP on EPI and EMT cells using the SNAI1 antibody to validate our *in silico* data. Our results show a consistently increased binding of SNAI1 (three-fold) to E-box 5, similar to E-cadherin used as a positive control (**Figure 3B**). Our results indicate that the CD73 gene is directly targeted by SNAI1 in MDA-MB-468 cells. This result agrees with a recently published report showing the direct binding of SNAI1 on CD73 promoter in mouse breast carcinoma cells (18).

Our data support that the regulation of CD73 in cells undergoing EMT occurs by direct binding of EMT-TFs to E-box motives in the *NT5E*/CD73 proximal promoter region. Among EMT-TFs, we identified SNAI1 as a major regulator of CD73 in TNBC. However, we cannot rule out that other EMT-TFs could also be involved in CD73 regulation in TNBC and other cancer types and settings. Consistent with this, it has been reported that EMT genomic signature is associated with *NT5E* expression in human HER2-positive breast tumors, and the EMT-TF TWIST was described to upregulate CD73 in immortalized mammary epithelial cells by a mechanism that is not fully understood (19). Another possible non-mutually exclusive mechanism by which EMT regulates the expression of CD73 is through EMT-dependent induction of cytokines such as TGF-β or TNF-α, as previously described (20, 21). Nevertheless, our data, together with previous reports, highlight the prominent role of EMT in CD73 immune checkpoint upregulation.

We next investigated whether a correlation between SNAI1 and CD73 expression is observed in TNBC patients. We first analyzed by qRT-PCR *NT5E*/CD73 and *SNAI1* gene expression in tumor mRNA from 12 TNBC patients. We observed a significant positive correlation between *NT5E*/CD73 and *SNAI1* expression (**Figure 3C**). We next validated our data using the large TNBC cohort described in the METABRIC dataset. We selected 258 TNBC patients based on their negative ER/PR/HER2 status (**Figure 3D**). Our results revealed a significant and positive correlation between *NT5E* and *SNAI1* and the mesenchymal marker *VIM* in the selected TNBC patients (**Figure 3E**). In contrast, *NT5E* was negatively correlated with the epithelial marker *CDH1* (**Figure 3E**). We also found that the expression of *SNAI1* and *VIM* is consistently high in TNBC samples displaying high *NT5E* levels (**Figure 3E**). These results support our data and strengthen the link between EMT and CD73 expression in TNBC. Data related to TNBC patients are provided in **Table S2**.

### SNAI1-dependent upregulation of CD73 increases the release of extracellular adenosine and mediates immunosuppression

**Figure 4:**
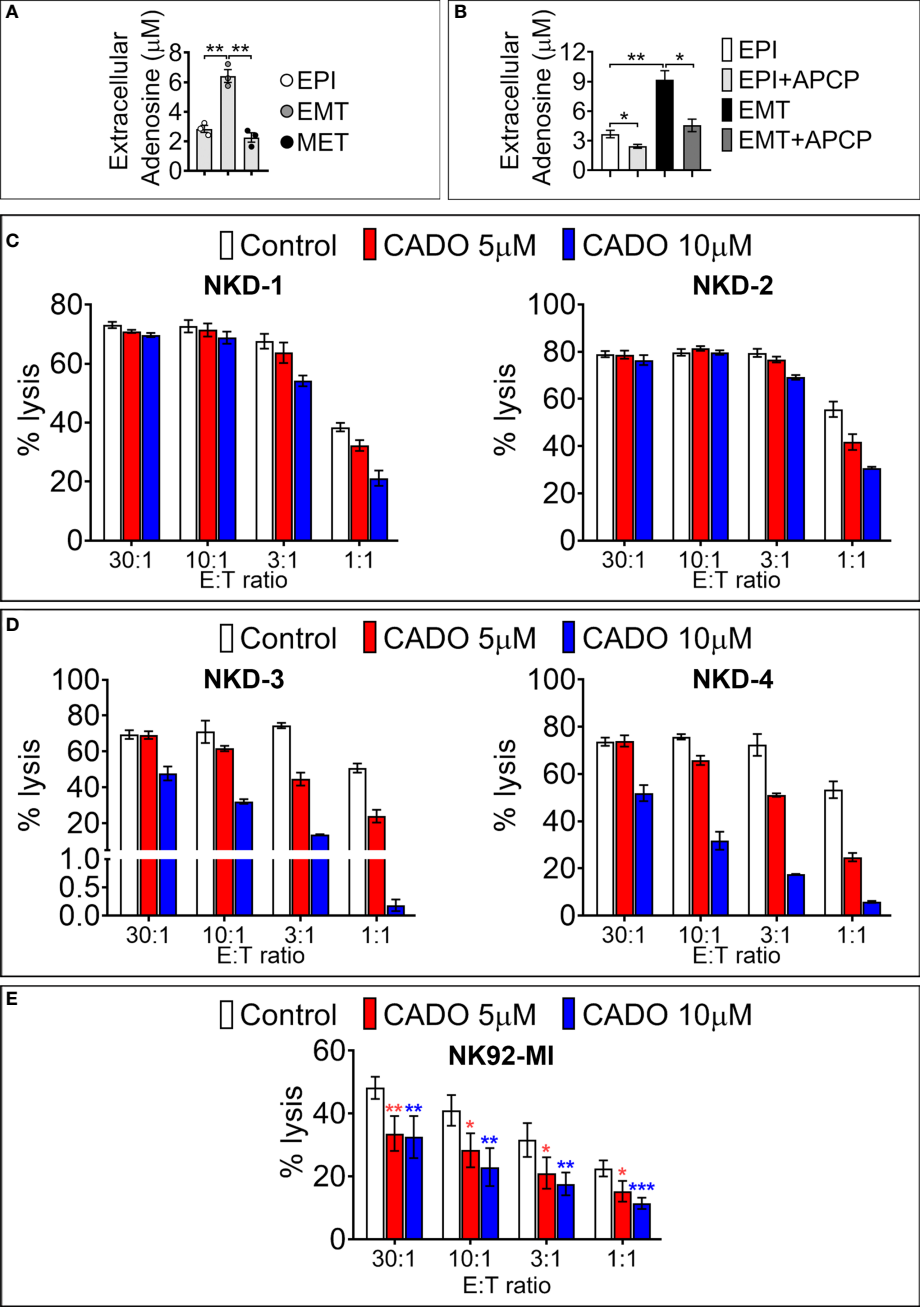
SNAI1-mediated CD73 upregulation results in increased extracellular ADO production. **(A)** Concentration of extracellular ADO released in the culture medium of MDA-MB-468-iSNAI1 cells cultured under control conditions (EPI), after 5 days of Dox (EMT), and after Dox removal (MET). Bars represent means from three independent experiments ± SEM (*P < 0.05, **P < 0.01 calculated by unpaired t-test). **(B)** Concentration of extracellular ADO released in the culture medium of MDA-MB-468-iSNAI1 cells cultured under control conditions (EPI) and after 5 days of Dox (EMT) in the absence or presence (+APCP) of APCP (100 µM). Bars represent means from three independent experiments ± SEM (*P < 0.05, **P < 0.01 calculated by unpaired t-test). **(C)** NK cells from four healthy donors (NKD-1 to 4) were pre-treated with CADO at 5 µM or 10 µM during 4 days (NKD-1 and NKD-2) or 6 days (NKD-3 and NKD-4). NK cytotoxic activity against K562 cells was measured at the indicated E:T ratios. Bars represent the mean percentage of lysis ± SD. **(D)** Cytotoxic activity of NK92-MI cells pre-treated for 6 days with CADO at 5 µM or 10 µM against K562 cells at the indicated **E:** T ratios. Bars represent mean percentage of lysis from three independent experiments ± SD (*P < 0.05, **P < 0.01, ***P < 0.001 calculated by unpaired t-test). Only one experiment was performed for each NK healthy donor reported in panels **(C, D)**. Experiments were performed either in duplicates or triplicates.

We next assessed the functional impact of SNAI1-dependent upregulation of cell surface CD73. We first analyzed the secreted ADO level in the conditioned medium of MDA-MB-468-iSNAI1 undergoing EMT. Extracellular ADO concentration was significantly increased from 2.82 ± 0.41 to 6.49 ± 0.74 µM following SNAI1-dependent upregulation of CD73 (EMT condition) and subsequently decreased to 2.17 ± 0.54 µM after Dox removal and SNAI1 and CD73 downregulation (MET condition) (**Figure 4A**). The increased release of extracellular ADO by MDA-MB-468-iSNAI1 cells cultured under EMT conditions was related to increased CD73 expression because such an increase was no longer observed following treatment of cells with the CD73 inhibitor Adenosine 5’-(α,β-methylene)diphosphate (APCP) (**Figure 4B**).

CD73 is considered a major source of intra-tumoral ADO production (22). Although its exact concentration in the tumor microenvironment is not yet well defined, it has been proposed that ADO concentration is in the micromolar range (23). In keeping with this, we next evaluated whether the level of ADO released following SNAI1-dependent EMT was sufficient to impair the cytotoxic properties of NK cells. The rationale for using NK cells relies on establishing a positive correlation between NK cell signature genes and TNBC patient survival (24). Intra-tumoral ADO elicits an immunosuppressive effect by interacting with Adenosine receptors. Indeed, four adenosine receptors have been identified including A_1_, A_2A_, A_2B_ and A_3_ (25, 26). The Adenosine A_2A_ receptor subtype is the predominant subtype found on T cells (27, 28) and NK cells (29).

We, therefore, analyzed the time- and concentration-dependent effects of the adenosine analog CADO on cytotoxic and proliferative capacities of NK cells isolated from four different healthy donors (NKD1, NKD2, NKD3, NKD4) and of the NK92-MI cell line. NKD1 and NKD2 were pre-treated for 4 days, whereas NKD3, NKD4, and NK92-MI were pre-treated for 6 days with CADO before co-culture with target cells at different effectors to target (E:T) ratios (30:1, 10:1, 3:1, and 1:1).

After 4 days of NK cell pre-treatment with 5 or 10 μM of CADO, we observed a decrease in NK-mediated lysis of target cells only at 3:1 and 1:1 E:T ratios (**Figure 4C**). After 6 days of NK cell pre-treatment with 5 or 10 μM of CADO, the impairment of NK-mediated lysis was observed at all E:T ratios tested except at the 30:1 ratio (**Figure 4D**). Similarly, pre-treatment of NK92-MI with 5 or 10 µM of CADO significantly impaired their cytotoxicity toward target cells at all E:T ratios tested (**Figure 4E**). The impairment of NK cell activity by CADO is associated with an impairment of their proliferation, as reported in **Figure S3**. Together, these results argue that ADO impairs the cytotoxic activity of NK cells in a time and dose-dependent manner and are in line with previous reports showing that adenosine analogs impair mouse NK cells’ mediated killing (30, 31).

### Concluding remarks

During the EMT process, the transcription factor SNAI1 acts as a direct repressor of E-cadherin promoter (32). In the present report we provide additional mechanistic insights showing that, similar to its role in CD47 upregulation (12), SNAI1-dependent EMT upregulates the expression of CD73 in TNBC cells. Furthermore, we show clinical evidence that such a regulation may occur in TNBC patients. Considering the encouraging but still moderate clinical responses of immune checkpoint blockades in TNBC, our results provide a rationale for further investigating the relevance of targeting EMT pathways in combination with immune checkpoint blockades to enhance the clinical benefit of immunotherapy in TNBC.

## Data availability statement

The original contributions presented in the study are included in the article/**Supplementary Material**. Further inquiries can be directed to the corresponding author.

## Author contributions

Conception and design: MH, MZ, and BJ. Development of methodology: MH, BH, MX, MZ, and BJ. Acquisition of data: MH, MX, KM and MZ. Analysis and interpretation of data: MH, AO, MM, MZ. Writing, reviewing, and/or revising the manuscript: MH, MZ and BJ. Administrative, technical, or material support: MV, BH, AC, MZ, GB and BJ. Study supervision: MH, CD, JT, MZ and BJ. All authors contributed to the article and approved the submitted version.

## Funding

This work was supported by the Luxembourg Institute of Health and grants from the FNRS Televie (grant 7.4579.20); Fondation Recherche Cancer et Sang (INCOM-BIOM), Luxembourg; Luxembourg National Research Fund (PRIDE15/10675146/CANBIO, C18/BM/12670304/COMBATIC and BRIDGES2021/BM/16358198/TRICK-ALDH); Fondation Cancer, Luxembourg (FC/2018/06); Kriibskrank Kanner Foundation, Luxembourg (2019); Janssen Cilag Pharma; Roche Pharma, Stiftelsen Cancera Sweden (2022) and Action LIONS Vaincre le Cancer Luxembourg (AB-2020 and MZN-2020).

## Acknowledgments

We thank Dr. Thomas Westbrook (Verna & Marrs McLean Department of Biochemistry & Molecular Biology, Baylor College of Medicine, Houston, TX) for the pINDUCER20 construct used to generate the MDA-MB-iSnail cell model.

## Conflict of interest

The authors declare that the research was conducted in the absence of any commercial or financial relationships that could be construed as a potential conflict of interest.

## Publisher’s note

All claims expressed in this article are solely those of the authors and do not necessarily represent those of their affiliated organizations, or those of the publisher, the editors and the reviewers. Any product that may be evaluated in this article, or claim that may be made by its manufacturer, is not guaranteed or endorsed by the publisher.

## Supplementary material

Figure S1 Epithelial and mesenchymal protein expression in MDA-MB-468 and MDA-MB-231 cells. Representative Western-blot analysis of three independent experiments showing protein levels of ZEB1, Vimentin, and E-cadherin in MDA-MB-468 vs. MDA-MB-231 cells. Actin was used as a loading control.

Figure S2 Expression of SNAI1 and CD73 mRNA in MDA-MB-468 cells treated with increasing doses of Dox. (A) RT-qPCR measurements of SNAI1 and CD73 mRNA using increasing doses of Dox, n=4. *P < 0.05 by Mann Whitney. Means ± SEM are shown. (B) Representative FACS contour plot of three independent experiments showing intra-cellular SNAI1 and surface CD73 staining in MDA-MB-468 iSNAI1 displaying EPI, EMT, and MET phenotype conditions. (C) Expression of epithelial and EMT markers (E-Cadherin, SNAIL1, ZEB1, N-Cadherin and Vimentin) in epithelial-like Py-230 and mesenchymal-like Py-8119 cells. Actin was loaded as a control. (D) The expression of full length (FL) and deleted (Del) forms of SNAIL1 in Py-8119 cells transfected with control (CTRL) or SNAIL1 (SNAIL1) CRISPR plasmids. Actin was loaded as a control. (E) RT-qPCR measurement of Nt5e mRNA in Py-8119 described in D. Nt5e expression was calculated relative to control cells expressing FL SNAIL1. Bars represent mean from four independent experiments ± SD; *P < 0.05 calculated by Mann Whitney. (F) Flow cytometry analysis of cell surface CD73 in cells described in D. Left panel: Delta mean fluorescence intensity (MFI); middle panel: percentage of CD73-positive (CD73+) cells and left panel: representative FACS histograms of indicated cells stained with control isotype or anti-CD73 antibody. Bars represent means from three independent experiments ± SD; *P < 0.05 calculated by unpaired t test.

Figure S3 Ki67 quantification in NK cells isolated from healthy donors (NKD-3 and -4) and NK92-MI cells following CADO treatment. Percentage of Ki67-positive cells in NKD-3, NKD-4, and NK92-MI after treatment for 6 days with CADO at 5 and 10 µM. For NK92-MI, bars represent the mean of three independent experiments ± SEM (*P < 0.05, ** P < 0.01 calculated by unpaired t-test)Table S1 Sequence of SYBR-GREEN RT-qPCR primers used for amplification of immunoprecipitated DNA samples from ChIP assays.

Table S1 Sequence of SYBR-GREEN RT-qPCR primers used for amplification of immunoprecipitated DNA samples from ChIP assays.

Table S2 Information about the TNBC patient described in Figure 3. The table shows the METABRIC 258 TNBC patient and sample IDs; sex; ER, HER2, PR, *NT5E* status, mRNA expression of *NT5E*, CDH1, SNAI1 and VIM. NP: Not profiled.
