# Supplementary figures and images for: SNAI1-dependent upregulation of CD73 increases extracellular adenosine release to mediate immune suppression in TNBC
Source: Front Immunol. 2022 Sep 9;13:982821. doi: 10.3389/fimmu.2022.982821 (PMC9501677; doi:10.3389/fimmu.2022.982821)

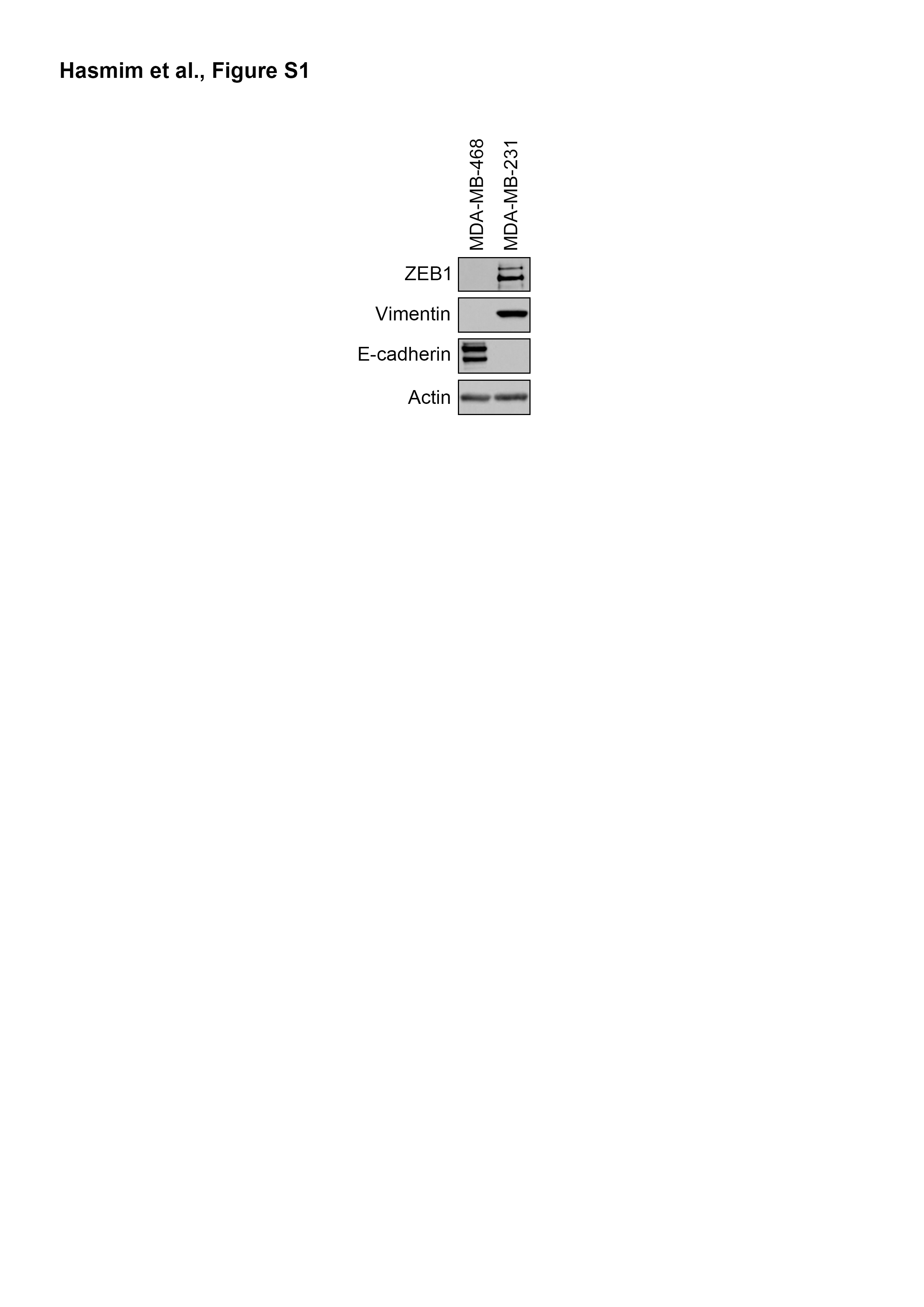

Supplement: Figure S1 — Epithelial and mesenchymal protein expression in MDA-MB-468 and MDA-MB-231 cells. Representative Western-blot analysis of three independent experiments showing protein levels of ZEB1, Vimentin, and E-cadherin in MDA-MB-468 vs. MDA-MB-231 cells. Actin was used as a loading control. [file Image_1.jpeg]

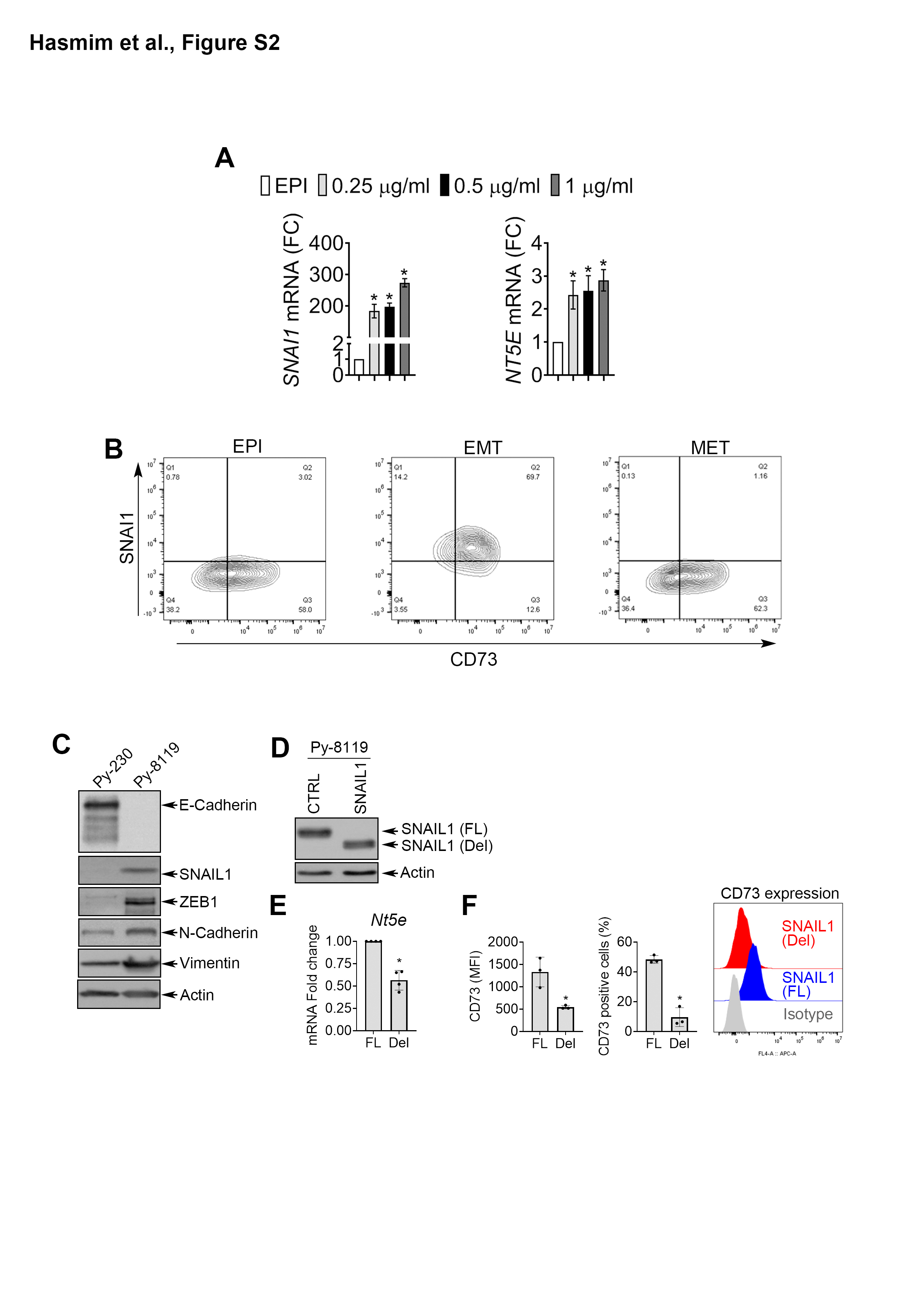

Supplement: Figure S2 — Expression of SNAI1 and CD73 mRNA in MDA-MB-468 cells treated with increasing doses of Dox. (A) RT-qPCR measurements of SNAI1 and CD73 mRNA using increasing doses of Dox, n=4. *P < 0.05 by Mann Whitney. Means ± SEM are shown. (B) Representative FACS contour plot of three independent experiments showing intra-cellular SNAI1 and surface CD73 staining in MDA-MB-468 iSNAI1 displaying EPI, EMT, and MET phenotype conditions. (C) Expression of epithelial and EMT markers (E-Cadherin, SNAIL1, ZEB1, N-Cadherin and Vimentin) in epithelial-like Py-230 and mesenchymal-like Py-8119 cells. Actin was loaded as a control. (D) The expression of full length (FL) and deleted (Del) forms of SNAIL1 in Py-8119 cells transfected with control (CTRL) or SNAIL1 (SNAIL1) CRISPR plasmids. Actin was loaded as a control. (E) RT-qPCR measurement of Nt5e mRNA in Py-8119 described in D. Nt5e expression was calculated relative to control cells expressing FL SNAIL1. Bars represent mean from four independent experiments ± SD; *P < 0.05 calculated by Mann Whitney. (F) Flow cytometry analysis of cell surface CD73 in cells described in D. Left panel: Delta mean fluorescence intensity (MFI); middle panel: percentage of CD73-positive (CD73+) cells and left panel: representative FACS histograms of indicated cells stained with control isotype or anti-CD73 antibody. Bars represent means from three independent experiments ± SD; *P < 0.05 calculated by unpaired t test. [file Image_2.jpg]

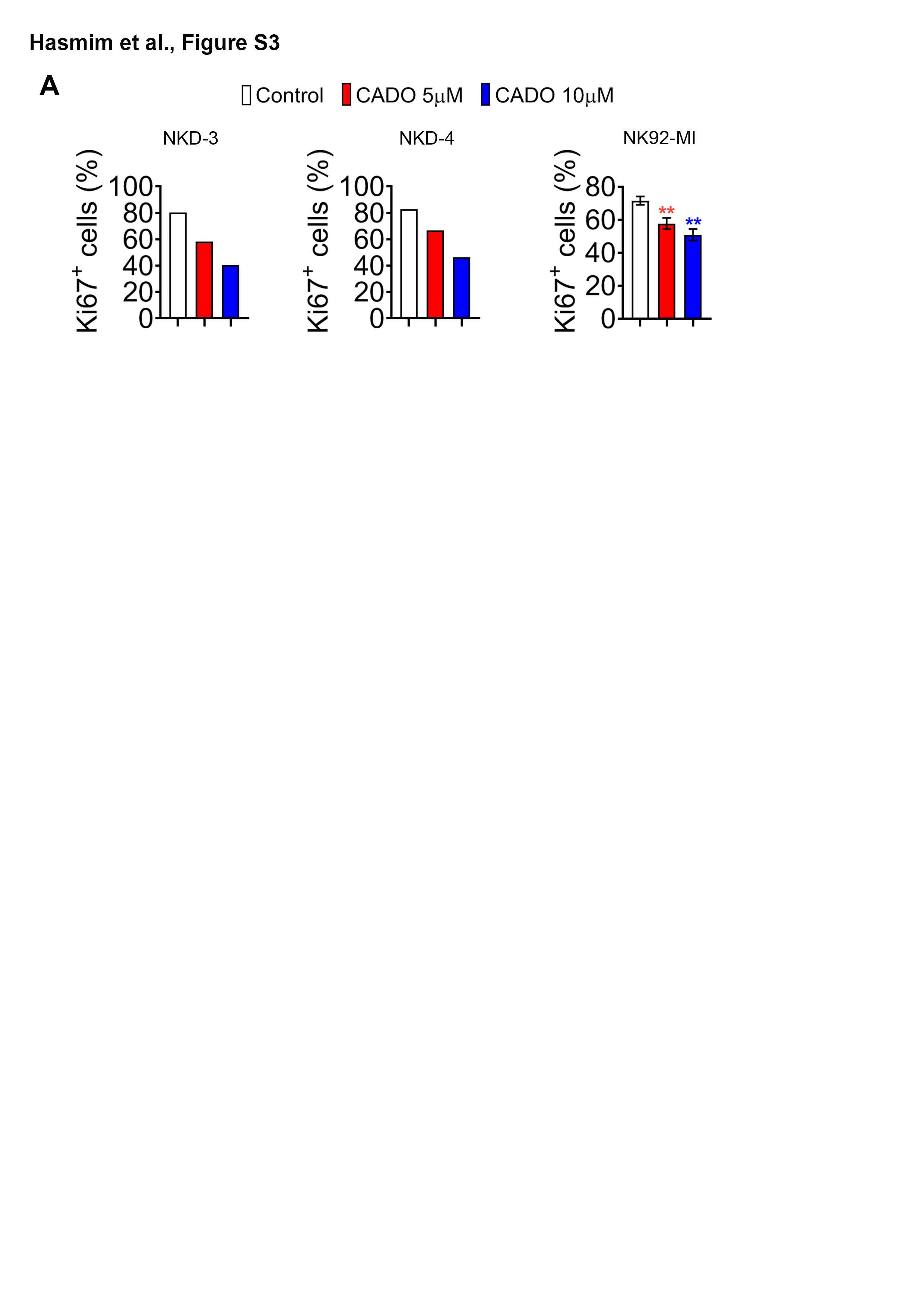

Supplement: Figure S3 — Ki67 quantification in NK cells isolated from healthy donors (NKD-3 and -4) and NK92-MI cells following CADO treatment. Percentage of Ki67-positive cells in NKD-3, NKD-4, and NK92-MI after treatment for 6 days with CADO at 5 and 10 µM. For NK92-MI, bars represent the mean of three independent experiments ± SEM (*P < 0.05, ** P < 0.01 calculated by unpaired t-test)Table S1 Sequence of SYBR-GREEN RT-qPCR primers used for amplification of immunoprecipitated DNA samples from ChIP assays. [file Image_3.jpeg]
